# Spatial Modulation and MP-WFRFT-Aided Multi-Beam Wireless Communication Scheme Based On Random Frequency Diverse Array

**DOI:** 10.3390/s20185289

**Published:** 2020-09-16

**Authors:** Jianbang Gao, Bin Qiu, Jing Zhou

**Affiliations:** 1 School of Electronic Engineering, Xi’an Shiyou University, Xi’an 710000, China; jzhou@xsyu.edu.cn; 2 School of Electronics and Information, Northwestern Polytechnical University, Xi’an 710072, China; qiubin@mail.nwpu.edu.cn

**Keywords:** physical layer security, spatial modulation, MP-WFRFT, cooperative LUs, power efficient

## Abstract

A security-enhanced, spectral-efficient, and power-efficient multi-beam wireless communication scheme based on random frequency diverse array (RFDA) is proposed in this paper. (AN)-aided directional modulation (DM) schemes. Furthermore, with the aid of spatial modulation (SM) technology and cooperative legitimate users (LUs), we can transmit more information bits by the use of LU number informations than the single modulation symbols. Unlike conventional zero-forcing (ZF) beamforming for multi-beam DM, we design a FDA beamforming vector for each LU based on the minimum transmit power method. Numerical simulations show that (1) the proposed scheme is power-efficient compared to the conventional schemes, (2) the proposed scheme can transmit more information bits than the conventional schemes, and (3) the proposed scheme can ensure communication security when eavesdroppers (Eves) are proximal to LUs or even in same locations.

## 1. Introduction

Wireless communication has attracted increasing attention in recent years. However, wireless communication occurs in an open environment and broadcasts information to all users in free space [1,2]. Therefore, wireless communication security has become a serious problem in both civil and military fields. Traditionally, upper-layer encryptions technology has been widely used in wired communication. However, encryption systems are inherited from the traditional computer network and ignores the special physical layer characteristics of wireless communication system, such as the openness wireless channel, time-varying network topology, and resource limitation of mobile terminal [3,4]. As a result, physical layer (PHY) security was introduced to achieve the confidentiality of messages at the PHY [5]. PHY security enables wireless communications to exploit the properties of physical layer to scramble information content that could be potentially intercepted by eavesdroppers, while simultaneously delivering it to its desired receivers. DM, as a keyless physical-layer security transmitting technique with great potentials, has attracted a great deal of attention over the past decade. It uses antenna arrays to transmit a signal only along the desired directions while distorting signal constellations in all other directions [6,7].

Traditionally, DM technology has been mainly implemented based on phased arrays (PA) in the past decade [8,9,10,11]. However, the previous communication schemes based on PA can no longer guarantee secure transmission when eavesdroppers locate in the desired direction of LU due to the transmit beampattern being only angle-focusing. Accordingly, it is necessary to investigate other schemes that can prevent eavesdroppers in the desired direction from intercepting messages. Therefore, we depart from PA and apply a frequency diverse array (FDA) into DM implementations because of its extra range dimension dependence, rather than being dependent on only the angle used [12,13,14,15,16,17].

FDA delivers a new opportunity for secure wireless communications. However, the beampattern of FDA is still angle-range coupling, which means that confidential messages are accessible to illegitimate receivers that are located at some angle-range pair curves. Several works have focused on frequency offsets to address the coupling problem of FDA [18]. To this end, in [19,20], the authors proposed a logarithmic frequency increments scheme. Furthermore, with random frequency increments between each element, the author of [21] proposed a new FDA structure, named random FDA, to indicate targets’ direction and range without coupling. Besides the radio frequency fronted technology, adding AN at the baseband is another effective technology used to deteriorate the received messages of Eves that has been employed in DM systems [22,23]. An FDA DM scheme with AN [24,25] has been proposed to further improve secrecy performance, and a robust synthesis scheme with AN was proposed in [26] for a single user scenario.

In summary, the current studies cannot deal with the PHY security problem of multiple receivers obtaining different messages simultaneously, which needs to be addressed in practical applications. The traditional DM schemes with AN need to allocate power to the AN, which will lower the power efficiency of total transmitting power. Furthermore, in practical cases, the Eves would be located as close to LU as possible (even in the same locations as the LUs) to eavesdrop on the confidential signal, and the previous studies have shown that it is difficult to ensure the security of the independent confidential message in this case. In addition, the received power at each LU is not accurately controlled according to the prescribed power.

In order to address the limitations of the previous works and further enhance the PHY security, we proposed a spatial modulation (SM) and multiple parameter weighted-type fractional Fourier transform (WFRFT)-aided scheme based on a random frequency diverse array (RFDA).

WFRFT, as a new transformation domain signal processing method based on Fourier transform [27], is gradually being applied to the wireless communication systems. WFRFT is actually the process of rotating the signal in the time-frequency plane to realize the time-frequency redistribution of signal power, and only when the users rotate the signal in same angle, but the opposite direction can the signal power be concentrated. Therefore, due to its power redistribution on the time-frequency plane, WFRFT technology can be regarded as a cryptographic method to further improve secrecy performance [28,29,30,31]. Furthermore, in [32], a synthesis scheme combining WFRFT and FDA DM was investigated to achieve power efficiency multi-beam secure communication. The contributions to physical layer security were promising. Apart from the above-mentioned WFRFT schemes based on a single parameter, the multiparameter WFRFT synthesis approaches have also been investigated intensively [33,34]. The MP-WFRFT system has a good parameter resistance, allowing it to detect in the condition of the eavesdroppers with a known signal transformation mode; in particular, this method can be combined with the existing DM technology, which can further improve the capacity for anti-interception and anti-detection based on the original system confidentiality.

SM, as an emerging information modulation technology, has gradually been introduced into wireless communication in recent years due to its high data rate and spectral efficiency. The basic idea of SM is to use the transmit antenna number as an additional information bearing unit to transmit more information bits than the single modulation symbols. However, in this paper, unlike previous works which applied SM to the multiple-input multiple-output (MIMO) system [35,36,37,38], the authors use SM technology based on FDA with cooperative LUs in order to avoid high interchannel interference at receivers and complicated estimate algorithms (e.g., maximum likelihood [35] and MRRC [38]) are required.

On the basis of the previous work, we propose a spectrally efficient and power-efficient multi-beam security communication scheme with the joint use of multiple techniques including MP-WFRFT, SM, and FDA–DM. Our main contributions can be summarized as follows.

(1) The proposed scheme combining MP-WFRFT realizes the embedding process of “AN” from the modulation level of digital baseband signal. Therefore, the proposed scheme based on MP-WFREFT avoids the power resource waste compared with traditional AN added scheme.

(2) In this paper, we apply SM technology into the FDA system with cooperative LUs, which can transmit additional information bits by using LUs number information compared to single modulation symbols to improve the capacity of communication system. Furthermore, the proposed scheme can ensure communication security when Eves are proximal to LUs or even in same locations.

(3) Unlike conventional beamforming method for multi-beam DM, we design the FDA beamforming matrix based on the minimum transmission messages power rule, which also can accurately control the received power of LUs.

The rest of this paper is organized as follows. Section 2 provides a review of DM PHY, MP-WFRFT and SM. Then, in Section 3, we propose the SM- and MP-WFRFT-aided scheme based on FDA. The performance is deduced in Section 4 and is numerically evaluated in Section 5. Finally, Section 6 draws conclusions.

## 2. Related Works

### 2.1. DM PHY

DM is a secure transmission technology without encryption for physical layer security. It uses antenna arrays to transmit confidential message only along desired directions while distorting signal constellations in all other directions. DM has received great attention in recent years, and it can be classified as PA-DM and FDA-DM. In this paper, we mainly research wireless communication scheme based on DM physical layer security technology.

PA-DM have been employed in many applications; however, these works based on PA-DM schemes can only achieve angle-dependent wireless physical layer security transmission [8,9,10]; Random subcarrier selection (RSCS) based on orthogonal frequency division multiplexing (OFDM) [11] only focuses on the single LU. Unless otherwise stated, other schemes are based on FDA-DM. The AN-aided DM schemes [14,22,23,24,25,26] can achieve the multi-beam secure transmission, regardless of the power efficiency. Moreover, those methods cannot achieve the neighbor security because of constraint on beamwidth. Furthermore, in [32], a synthesis scheme combined WFRFT and FDA DM was investigated to achieve power efficiency and the neighbor security. The contributions to physical layer security are solid. However, WFRFT DM schemes cannot guarantee the security of confidential messages when the WFRFT parameters are leaked to Eves.

On the basis of the previous work, we propose a synthesis multi-beam security communication scheme with the joint use of multiple techniques including MP-WFRFT, SM, and FDA-DM. Moreover, there are four potential practical applications for the proposed schemes in free space.

The first is the secure transmissions of satellite communications (SatCom) from ground station to satellites or from one satellite to others. The second is the secure transmissions of unmanned aerial vehicles (UAV) from the ground controller to UAVs or from one UAV to others. The third is the secure 5G millimeter wave (mmWave) communication. We can ignore the very few multi-path components in mmWave transmission, and the far-field and LoS assumptions can hold simultaneously due to the tiny array size, usually in magnitude of millimeters. The fourth is application in the Internet of Things (IoT). IoT is an indispensable part of our lives. Traditional security mainly relies on key encryption mechanism, but for the IoT with large number of nodes and heterogeneous networks, it is difficult to extract, distribute, and manage the keys. This has led us to develop new security solutions for IoT applications. Our proposed synthesis scheme based on FDA-DM can achieve security of IoT in physical layer, and it can solve the problem of spectrum scarcity in the IoT due to the SM technology.

### 2.2. MP-WFRFT

MP-WFRFT, known as a variation of the Fourier transform, is essentially the weighted sum of four basis functions. MP-WFRFT can be regarded as the process of rotating the signal in the time–frequency plane to realize the time–frequency redistribution of signal power. Only when the users rotate the signal in the same angle but the opposite direction can the signal power be concentrated. The representation of the 4-WFRFT of discrete sequences was proposed in [27] and has been widely employed in communication systems. Therefore, the classical 4-MP-WFRFT approach is adopted in this paper. For an arbitrary complex sequence $x={\left[x_{1},x_{2},\cdots ,x_{Q}\right]}^{T}$, the corresponding MP-WFRFT can be defined as

(1) $$\begin{array}{c} \begin{array}{c} F_{\alpha }^{\left(m,c\right)}\left(x\right)=\omega_{0}x+\omega_{1}F\left(x\right)+\omega_{2}F^{2}\left(x\right)+\omega_{3}F^{3}\left(x\right), \end{array} \end{array}$$

where F denotes the transform operator of MP-WFRFT; F, $F^{2}$, and $F^{3}$ denote 1–3 times discrete Fourier transform (DFT) of sequence x, respectively; α is the MP-WFRFT modulation order; $m={\left[m_{0},m_{1},m_{2},m_{3}\right]}^{T}$ and $c={\left[c_{0},c_{1},c_{2},c_{3}\right]}^{T}$ are integer vectors; and the weighting coefficient $\omega_{l}\left(l=0,1,2,3\right)$ is defined as

(2) $$\begin{array}{c} \begin{array}{c} \omega_{l}=\frac{1}{4}\sum_{k=0}^{3}\exp\{\frac{2\pi j}{4}\left[\alpha \left(4m_{k}+1\right)\left(4c_{k}+k\right)-lk\right]\}, \end{array} \end{array}$$

Based on the principles of transform operator F, $F^{2}\left(x\right)=\mathrm{Px}$, $F^{3}\left(x\right)=PF\left(x\right)$, where P is the Q×Q reverse matrix. Therefore, (2) can be expressed as

(3) $$\begin{array}{c} \begin{array}{c} F_{\alpha }^{\left(m,c\right)}\left(x\right)=\omega_{0}x+\omega_{1}F\left(x\right)+\omega_{2}\mathrm{Px}+\omega_{3}PF\left(x\right), \end{array} \end{array}$$

Furthermore, MP-WFRFT satisfies additive property, which can be expressed as

(4) $$\begin{array}{c} \begin{array}{c} F_{\alpha +\beta }^{\left(m,c\right)}\left(x\right)=F_{\alpha }^{\left(m,c\right)}\left[F_{\beta }^{\left(m,c\right)}\left(x\right)\right], \end{array} \end{array}$$

where α,β∈R are real numbers.

**Figure 1:**
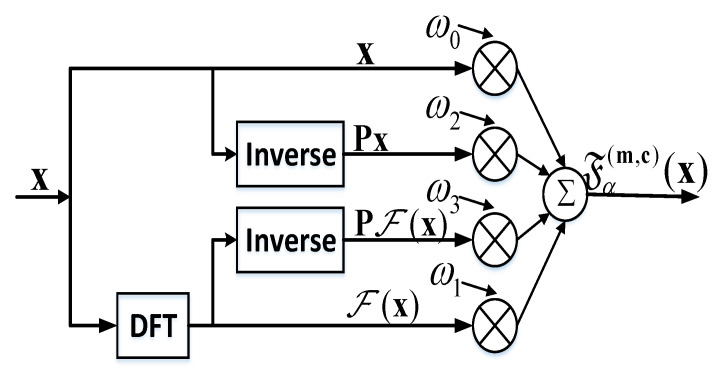
The implementation of the MP-WFRFT.

The implementation of the MP-WFRFT operation is demonstrated in Figure 1. It is obvious that the MP-WFRFT can be quickly realized by means of an inversion module and DFT module. Furthermore, from (4), the original sequence for 4-MP-WFRFT can be easily recovered only under the premise that the WFRFT parameter α is substituted with −α, i.e., $x=F_{-\alpha }^{\left(m,c\right)}\left[F_{\alpha }^{\left(m,c\right)}\left(x\right)\right]$. Therefore, the MP-WFRFT technique provides encryption security with parameters (α,m,c). LUs can decode confidential messages by using perfect MP-WFRFT parameters, and Eves only receive a distorted signal that is equivalent to noise.

### 2.3. SM Based On FDA

SM, as an emerging information modulation technique, has gradually been used in wireless communication in recent years. The basic idea of SM is to exploit spatial location information to transmit messages. Traditional SM is to map block of upcoming information based on transmit antenna number carrying unit and constellation diagram carrying unit (e.g., M-PSK). However, in this paper, we research wireless communication based on FDA with cooperative LUs. Therefore, unlike the traditional SM technique, we apply SM into FDA by directly using LUs number information as a carrying unit to transmit more information bits. At the receiving end, one or some LUs will be active and receive messages. The other LUs are inactive and receive zero power. In our scheme, only if we correctly estimate the LUs number informations and the received symbols will the block of information bits retrieved by the cooperative LUs. We achieve security communication in the case Eves are proximal to LUs or even in same locations.

In general, our proposed scheme maps the block of information bits, which is determined by the number of LUs and digital modulation. The size of each block for a system that use M-PSK or M-QAM constellation diagram and K LUs can be calculated as

(5) $$\begin{array}{c} \begin{array}{c} B=\log_{2}\left(K\right)+Q\log_{2}\left(M\right), \end{array} \end{array}$$

where the first term $\log_{2}\left(K\right)$ is the number of information bits carried by LUs number. *Q* is the number of active LUs, and the second term $Q\log_{2}\left(M\right)$ is the number of information bits carried by symbols of constellation diagram, which are received by *Q* active LUs, respectively. For example, we set the BPSK constellation diagram (M=2), the number of LUs is K=4, and the number of active is Q=3, which maps five message bits as a block to be transmitted at one time, as shown in Table 1.

## 3. SM and MP-WFRFT Aided Scheme Based On FDA

### 3.1. The Architecture of System with Cooperative LUs

**Figure 2:**
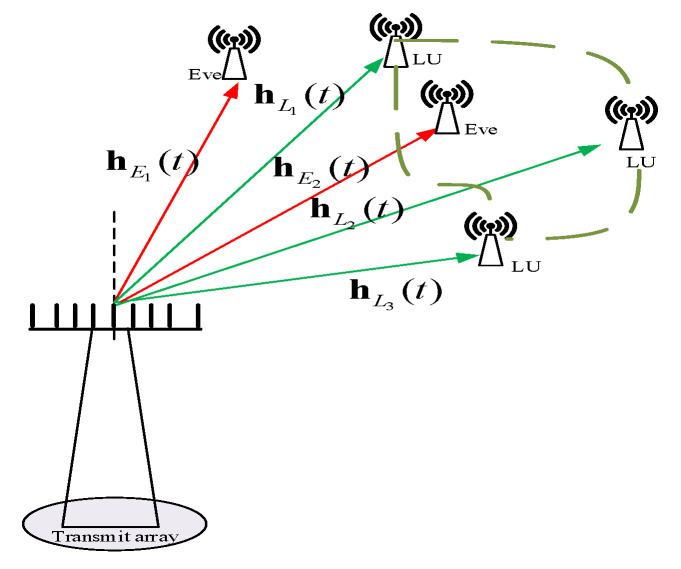
System model.

The traditional PA-DM schemes and even the AN-aided PA-DM synthesis schemes cannot achieve range dependence, and so these schemes cannot prevent eavesdroppers from intercepting messages and recognize private users in same direction. Therefore, we depart from PA and apply FDA into our DM implementations because of its extra range dimension dependence rather than simply angle dependence. In this paper, the line-of-sight (LoS) channel, far-field communication, and Gaussian wiretap channel are considered. As shown in Figure 2, the system consists of a legitimate transmit station, *K* LUs whose information can be shared with each other, and *J* passive Eves whose locations are unavailable to transmitter station.

A uniform *N* elements linear array with a spacing *d* is utilized for the transmitter. The transmitting frequency at the n−th (n = 1, 2, …, N) antenna is designed as $f_{n}=f_{c}+\Delta f_{n}$, where $f_{c}$ is the carrier wave frequency and $\Delta f_{n}$ is the frequency increment. Here, we propose RFDA whose beam pattern is angle-range independent without coupling. Therefore, $\Delta f_{n}$ can be replaced as $\Delta f_{n}=\lambda_{n}\Delta f$, where Δf refers to a fixed frequency increment, and $\lambda_{n}$ represents a random variable.

For an arbitrary receiver at (r,θ), the normalized steering vector is denoted by

(6) $$\begin{array}{c} \begin{array}{c} h\left(\theta ,r,t,f\right)=\frac{\rho (r)}{\sqrt{N}}\left[e^{-j2\pi f_{1}\left(t-\frac{r}{c}\right)},e^{-j2\pi f_{2}\left(t-\frac{r-d\sin\theta }{c}\right)}\right.{\left.,\ldots ,e^{-j2\pi f_{N}\left(t-\frac{r-(N-1)d\sin\theta }{c}\right)}\right]}^{T} \end{array} \end{array}$$

where *c* denotes light speed, ρ(r) refers to the path loss factor due to the free space propagation.

The location of LU *K* is $\left(r_{l_{k}},\theta_{l_{k}}\right)$, and for simplicity $h{\left(t\right)}_{L_{k}}\overset{\Delta }{\;=}h_{L_{k}}\left(r,\theta ,t,f\right)$ is the normalized steering vector of LU *K*. Furthermore, we use the steering matrix $H_{L}\left(t\right)$ to denote steering vectors of all LUs, i.e.,

(7) $$\begin{array}{c} \begin{array}{c} H_{L}\left(t\right)\overset{\Delta }{\;=}\left[h_{L_{1}}\left(t\right),h_{L_{2}}\left(t\right),\cdots ,h_{L_{k}}\left(t\right),\cdots ,h_{L_{K}}\left(t\right)\right], \end{array} \end{array}$$

where $h_{L_{k}}\left(t\right)$ is the instantaneous normalized steering vector of *k*-th LU at $\left(r_{L_{k}},\theta_{L_{k}}\right)$.

### 3.2. The Radiating Signal Processed by SM and MP-WFRFT

**Figure 3:**
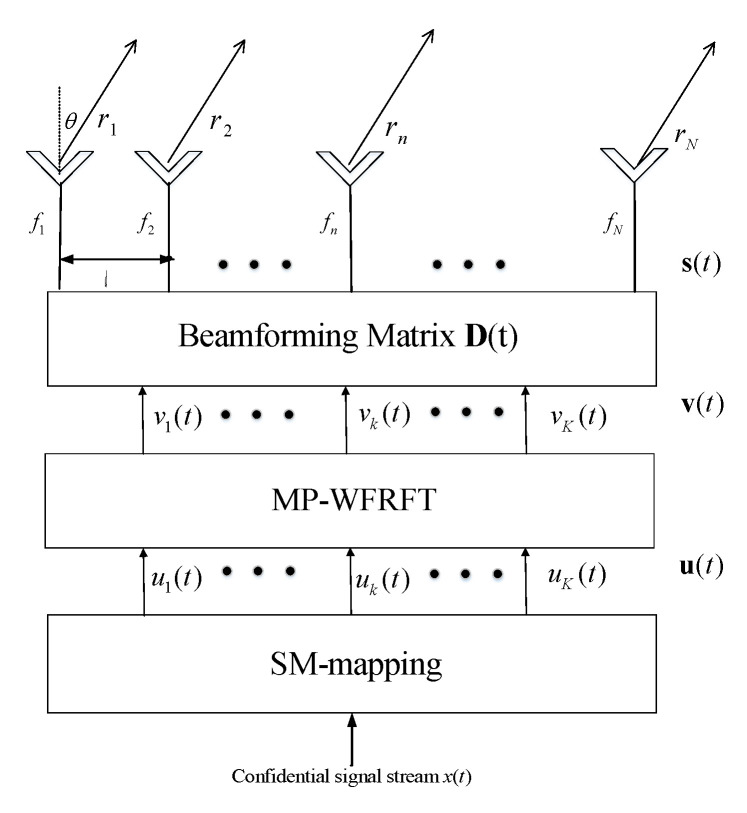
The architecture of the transmit station.

The architecture of transmit station is shown in Figure 3. In this paper, we innovatively apply two key modulation techniques into FDA-DM. Before transmitting the confidential signal to LUs, we first use the SM module for the confidential signal stream x(t) and x(t) is normalized, i.e., $E[{\left|x(t)\right|}^{2}]=1$. Then, we divide the confidential information bit stream into blocks. As mentioned in previous section, each block contains $\log_{2}\left(KM^{Q}\right)$ bits. The SM is operated to the confidential information block, which yields the transmitting symbol vector

(8) $$\begin{array}{c} \begin{array}{c} u\left(t\right)=U\left[x\left(t\right)\right]={\left[u_{1}\left(t\right),u_{2}\left(t\right),\ldots ,u_{K}\left(t\right)\right]}^{T}, \end{array} \end{array}$$

where $u_{k}\left(t\right)$ is the transmitting symbol for the *k*-th LU, and U[·] is the SM mapping.

Second, the transmitting symbol vector u(t) is performed by MP-WFRFT with parameters ${\left[\alpha_{s},m_{s},c_{s}\right]}^{T}$, which can be expressed as

(9) $$\begin{array}{c} \begin{array}{c} v=F_{\alpha_{s}}^{\left(m_{s},c_{s}\right)}\left(u\right)=w_{0}u+w_{1}F\left(u\right)+w_{2}\mathrm{Pu}+w_{3}PF\left(u\right), \end{array} \end{array}$$

The vector u(t) is rotated in the time-frequency plane to realize the time-frequency redistribution of signal power. Therefore, only when the users rotates the signal in same angle but the opposite direction can the signal power be concentrated.

Before radiating, we design the beamforming matrix D to further process the symbol vector v to match all transmit antennas. The beamforming matrix D is given by

(10) $$\begin{array}{c} \begin{array}{c} D=[d_{1}\left(t,f\right),d_{2}\left(t,f\right),\ldots ,d_{K}\left(t,f\right)], \end{array} \end{array}$$

where $d_{k}\left(t,f\right)={\left[d_{k,1}\left(t,f\right),d_{k,2}\left(t,f\right),\ldots ,d_{k,N}\left(t,f\right)\right]}^{T}$, for k=1,2…,K, is the beamforming vector to process symbol $v_{k}\left(t\right)$ that is transmitted to *k*-th LU.

In order to obtain the beamforming matrix D, the locations of LUs are assumed to be previously known by transmit station, i.e., the steering matrix $H_{L}\left(t\right)$ is known in advance. Next, we design the beamforming matrix D based on the rules that (1) the intended LU effectively receives the corresponding confidential messages, while the undesired LUs cannot obtain the messages, and (2) the transmit power is minimum, while satisfying communication performance requirements of each LU. Therefore, the beamforming matrix D is designed by

(11) $$\begin{array}{c} \begin{array}{c} H_{L}^{H}\left(t\right)D=AI_{K}, \end{array} \end{array}$$

where A=diag[ζ], in which $\zeta \overset{\Delta }{\;=}[\sqrt{\zeta_{1}},\sqrt{\zeta_{2}},\ldots ,\sqrt{\zeta_{k}},\ldots ,\sqrt{\zeta_{K}}]$, and $\zeta_{k}$ is the minimum desired power, for k=1,2,⋯,K.

According to the pseudo-inverse concept, we obtain D as

(12) $$\begin{array}{c} \begin{array}{c} D=H_{L}\left(t\right){\left(A^{-1}\right)}^{H}{\left[A^{-1}H_{L}^{H}\left(t\right)H_{L}\left(t\right){\left(A^{-1}\right)}^{H}\right]}^{-1}, \end{array} \end{array}$$

After processed by the beamforming matrix, the radiating signal for the *N* antennas is given by

(13) $$\begin{array}{c} \begin{array}{c} s=\mathrm{Dv}=DF_{a_{s}}^{\left(m_{s},c_{s}\right)}\left[U(x\right)], \end{array} \end{array}$$

### 3.3. The Received Signal of LUs and Eves

**Figure 4:**
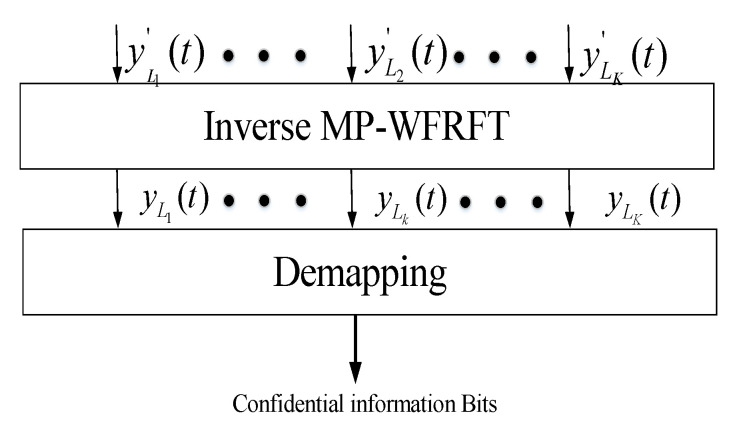
The receiving structure with cooperative legitimate users (LUs).

The receiving structure with cooperative LUs is shown in Figure 4. Because of cooperative LUs, we combine all LUs received signals together as an LUs received vector, i.e., $y_{L}\left(t\right)={\left[y_{L_{1}}\left(t\right),y_{L_{2}}\left(t\right),\ldots ,y_{L_{K}}\left(t\right)\right]}^{T}$. In this paper, assuming that (1) the synchronization of time and frequency is perfect in the ideal scenario, and (2) the MP-WFRFT parameters are securely shared between the transmit station and LUs. As shown in Figure 4, the received symbol is first operated by inverse MP-WFRFT with $\left(\alpha_{l},m_{l},c_{l}\right)$, which yields

(14) $$\begin{array}{c} \begin{array}{c} y_{L}\left(t\right)=F_{\alpha_{l}}^{\left(m_{l},c_{l}\right)}\left(y_{L}^{{}^{\prime }}\left(t\right)\right)=F_{\alpha_{l}}^{\left(m_{l},c_{l}\right)}\left(H_{L}^{H}\left(t\right)s\left(t\right)\right)+F_{\alpha_{l}}^{\left(m_{l},c_{l}\right)}\left(n_{L}\left(t\right)\right) \end{array} \end{array}$$

where the shared parameters $\left(\alpha_{l},m_{l},c_{l}\right)=\left(-\alpha_{s},m_{s},c_{s}\right)$. Based on (9), (11), and (13), (14) can be further simplified as

(15) $$\begin{array}{c} \begin{array}{cc} y_{L}\left(t\right) & =F_{-\alpha_{s}}^{\left(m_{s},c_{s}\right)}\left(\mathrm{Av}\left(t\right)\right)+F_{-\alpha_{s}}^{\left(m_{s},c_{s}\right)}\left(n_{L}\left(t\right)\right)=AF_{-\alpha_{s}}^{\left(m_{s},c_{s}\right)}F_{\alpha_{s}}^{\left(m_{s},c_{s}\right)}\left(u\left(t\right)\right)+F_{-\alpha_{s}}^{\left(m_{s},n_{s}\right)}\left(n_{L}\left(t\right)\right)\\ & =\mathrm{Au}\left(t\right)+n_{L}^{{}^{\prime }}\left(t\right) \end{array} \end{array}$$

where $n_{L}\left(t\right)={\left[n_{L_{1}}\left(t\right),n_{L_{2}}\left(t\right),\ldots ,n_{L_{k}}\left(t\right),\ldots ,n_{L_{K}}\left(t\right)\right]}^{T}$ is the AWGN vector with each element having zero mean and variance $\sigma_{L_{k}}^{2}$, i.e., $n_{L}\left(t\right)∼\mathrm{CN}\left(0_{K\times 1},\sigma_{L}^{2}I_{K}\right)$. $n_{L}^{{}^{\prime }}\left(t\right)={\left[n_{L_{1}}^{{}^{\prime }}\left(t\right),n_{L_{2}}^{{}^{\prime }}\left(t\right),\ldots ,n_{L_{k}}^{{}^{\prime }}\left(t\right),\ldots ,n_{L_{K}}^{{}^{\prime }}\left(t\right)\right]}^{T}$ is the AWGN vector after MP-WFRFT, which remains the same distribution characteristics, i.e., $n_{L}^{{}^{\prime }}\left(t\right)∼\mathrm{CN}\left(0_{K\times 1},\sigma_{L}^{2}I_{K}\right)$. Thereinto, the received symbol of *k*-th LU is given by

(16) $$\begin{array}{c} \begin{array}{cc} y_{L_{k}}\left(t\right) & =F_{-\alpha_{s}}^{\left(m_{s},c_{s}\right)}\left(h_{L_{k}}^{H}\left(t\right)s\left(t\right)\right)+n_{L_{k}}^{{}^{\prime }}\left(t\right)=h_{L_{k}}^{H}\left(t\right)d_{k}\left(t\right)u_{k}\left(t\right)+h_{L_{k}}^{H}\left(t\right)\sum_{i=1,i\neq k}^{K}d_{i}\left(t\right)u_{i}+n_{L_{k}}^{{}^{\prime }}\left(t\right)\\ & =\sqrt{\zeta_{k}}u_{k}\left(t\right)+n_{L_{k}}^{{}^{\prime }}\left(t\right) \end{array} \end{array}$$

From (15) and (16), it can be seen that each LU can effectively receive the corresponding transmitting symbol under the control of desired received power, and the transmitting symbol vector u(t) is recovered via inverse MP-WFRFT with cooperative LUs. Then, after the correct reception and inverse MP-WFRFT of all LUs, the confidential signal stream x(t) is obtained by demapping the transmitting symbol vector u(t).

Next, we assume there are *J* passive Eves located in different positions intercepting the confidential information. We define $\left(r_{e_{j}},\theta_{e_{j}}\right)$ as the coordinates of Eve *j* and use the steering matrix $H_{E}\left(t\right)$ to denote steering vectors of all Eves, i.e.,

(17) $$\begin{array}{c} \begin{array}{c} H_{E}\left(t\right)\overset{\Delta }{\;=}\left[h_{E_{1}}\left(t\right),h_{E_{2}}\left(t\right),\cdots ,h_{E_{j}}\left(t\right),\cdots ,h_{E_{J}}\left(t\right)\right], \end{array} \end{array}$$

where $h_{E_{j}}\left(t\right)$ is the instantaneous normalized steering vector of *j*-th Eve. Furthermore, we consider a worse case in which Eves in different positions can cooperate with each other. Similarly, the received message of Eves is given by

(18) $$\begin{array}{c} \begin{array}{c} y_{E}\left(t\right)=H_{E}^{H}\left(t\right)D\left(t\right)v\left(t\right)+n_{E}\left(t\right)=\underset{\mathrm{Scrambled}\;\mathrm{signal}}{\underset{︸}{H_{E}^{H}\left(t\right)D\left(t\right)w_{0}u\left(t\right)}}+\underset{\mathrm{Equivalant}\;\mathrm{AN}}{\underset{︸}{H_{E}^{H}\left(t\right)D\left(t\right)b}}+\underset{\mathrm{AWAG}}{\underset{︸}{n_{E}\left(t\right)}} \end{array} \end{array}$$

where $b=w_{1}F\left(u\right)+w_{2}\mathrm{Pu}+w_{3}PF\left(u\right)={\left[b_{1}\left(t\right),b_{2}\left(t\right),\ldots ,b_{K}\left(t\right)\right]}^{T}$, and $n_{E}\left(t\right)$ is the AWGN vector with each element having zero mean and variance $\sigma_{E_{j}}^{2}$, i.e., $n_{E}\left(t\right)∼\mathrm{CN}\left(0_{J\times 1},\sigma_{E}^{2}I_{J}\right)$. Specifically, the received signal of *j*-th Eve intercepting *k*-th LU’s information is given by

$$\begin{array}{cc} y_{E_{j,k}} & =h_{E_{k}}^{H}\left(t\right)d_{k}\left(t\right)v\left(t\right)+n_{E_{k}}\left(t\right)\\ & =\underset{\mathrm{Scrambled}\;\mathrm{signal}}{\underset{︸}{h_{E_{k}}^{H}\left(t\right)d_{k}\left(t\right)w_{0}u_{k}\left(t\right)}}+\underset{\mathrm{Interference}\;\mathrm{from}\;\mathrm{others}}{\underset{︸}{h_{E_{k}}^{H}\left(t\right)\sum_{i=1,i\neq k}^{K}d_{i}\left(t\right)w_{0}u_{i}}}+\underset{\mathrm{Equivalant}\;\mathrm{AN}}{\underset{︸}{h_{E_{k}}^{H}\left(t\right)\sum_{i=1}^{K}d_{i}\left(t\right)b_{i}}}+\underset{\mathrm{AWAG}}{\underset{︸}{n_{E_{k}}\left(t\right)}} \end{array}$$

according to the first part of (18), the amplitude and phase of the received symbol is distorted by MP-WFRFT operations $w_{0}$ and the item $H_{E}^{H}\left(t\right)D\left(t\right)\notin R$. The second part of (18) mainly shows interference from other messages, and the third part is the equivalent AN as the process of rotating the signal in the time-frequency plane due to MP-WFRFT. The last part is AWGN. Our proposed scheme does not add noise into the baseband signal, but the application of MP-WFRFT in our proposed method also can achieve an equivalent noise effect on Eves, which means it uses less power compared to conventional AN-DM schemes.

On the other hand, MP-WFRFT technology is dependent on the assumption that the MP-WFRFT parameters are unknown for Eves. Once the MP-WFRFT parameters are leaked to Eves, Eves will demodulate their received signals via inverse MP-WFRFT operation. However, in this paper, we use another SM technique based on FDA with cooperative LUs. After the use of the SM and MP-WFRFT aided multi-beam FDA scheme, it is hard for Eves to wiretap the confidential messages. The Eves can correctly recover the confidential messages, only when estimates of LUs number information, the MP-WFRFT parameters and the corresponding symbols are all correct. Furthermore, even if one or some (but not all) of the Eves’ locations are same as one or some LUs’ locations and MP-WFRFT parameters are leaked to all Eves, our proposed scheme can also ensure the security wireless communication due to the use of SM technique with cooperative LUs. Therefore, our proposed method is able to degrade Eves’ reception and improve transmission security. Furthermore, our proposed method can achieve power efficient due to MP-WFRFT technology and information bits efficient by use of LUs number information as another information carrying unit in addition to constellation diagram.

## 4. Performance Analysis

With the basic knowledge of the SM and MP-WFRFT aided RFDA-DM scheme, we next analyze the secrecy performance of the system through the symbol error rate (SER) and bit error rate (BER), which are important metrics to measure the performance of wireless communication systems. Moreover, we analyze the anti-interception performance and provide comparisons with different DM schemes.

### 4.1. Symbol Error Rate

The confidential messages can be recovered only when estimates of MP-WFRFT parameters, the number information of LUs, and the corresponding modulation symbols are all correct. Here, we consider that the MP-WFRFT parameters are securely shared between transmitter station and LUs. Therefore, in order to calculate the overall SER $P_{s}$, we should consider the probability of error $P_{a}$ for the estimates of number information and the SER $P_{m}$ of corresponding modulation symbols. The overall SER $P_{s}$ can be calculated as

(19) $$\begin{array}{c} \begin{array}{c} P_{s}=1-\left(1-P_{a}\right)\left(1-P_{m}\right). \end{array} \end{array}$$

First, the $P_{m}$ of corresponding modulation symbols is calculated. Only BPSK modulation, i.e., M=2, is considered throughout this paper. Moreover, the number of active LU is Q=2. The theoretical SER $P_{m}$ over AWGN channel can be obtained by

(20) $$\begin{array}{c} \begin{array}{c} P_{m}=1-{\left(1-Q\left({\sqrt{r}}_{k}\right)\right)}^{2}=2Q\left({\sqrt{r}}_{k}\right)-Q^{2}\left({\sqrt{r}}_{k}\right), \end{array} \end{array}$$

where $Q\left(x\right)=\frac{1}{\sqrt{2\pi }}\int_{x}^{\infty }\exp(-\frac{x^{2}}{2})dx$ is the complementary error function. $r_{k}$ is the signal to interference-plus-noise ratio (SINR). According to Equation (16), we can calculate the $r_{k}$ of LU *k* as

(21) $$\begin{array}{c} \begin{array}{c} r_{k}=\frac{\zeta_{k}}{\sigma_{L}^{2}}. \end{array} \end{array}$$

According to the Equations (10), (11), and (12), the total transmit power $P_{s}$ can be calculated as

(22) $$\begin{array}{c} \begin{array}{c} P_{s}=\sum_{i=1}^{K}{∥d_{k}\left(t,f\right)∥}_{2}^{2}. \end{array} \end{array}$$

By contrast, with the conventional AN-DM shceme, we need allocate power to AN to suppress the signal received by Eve. Therefore, the transmit power of confidential message can be expressed as $P_{m}=\beta^{2}P_{s}$, and the SINR $r_{k}$ of LU *k* can be obtained by

(23) $$\begin{array}{c} \begin{array}{c} r_{k}^{AN}=\frac{\beta^{2}\zeta_{k}}{\sigma_{L}^{2}}=\beta^{2}r_{k}. \end{array} \end{array}$$

where β is the power splitting coefficient for confidential messages.

According to the Equation (19), we can calculate the $r_{k}$ of *j*-th Eve intercepting *k*-th LU’s information as

$$\begin{array}{c} r_{k}=\frac{{\left|h_{E_{j}}^{H}\left(t\right)d_{k}\left(t\right)\right|}^{2}}{\sum_{i=1,i\neq k}^{K}{\left|h_{E_{j}}^{H}\left(t\right)d_{i}\left(t\right)w_{0}\right|}^{2}+\sum_{i=1}^{K}{\left|h_{E_{j}}^{H}\left(t\right)d_{i}\left(t\right)b_{i}\right|}^{2}+\sigma_{E}^{2}}. \end{array}$$

Next, the probability error $P_{a}$ at LUs and Eves is calculated, respectively. Here, we consider the probability error $P_{a}$ for the LUs. In this paper, we assume that the LUs know the SM mapping in advance and that Eves cannot wiretap any SM information. Meanwhile, each independent LU effectively receives the confidential signal, which is verified in the next subsection. Therefore, we consider a reasonable approximation $P_{a}\approx 0$ at LUs. Then, we calculate $P_{a}$ at Eves. Due to the arbitrary locations, Eves cannot wiretap any number information. Therefore, $P_{a}$ at Eves can be obtained by

(24) $$\begin{array}{c} \begin{array}{c} P_{a}=1-\frac{1}{2^{K}-1}. \end{array} \end{array}$$

### 4.2. Anti-Interception Performance

In this subsection, the anti-interception performance of the proposed MP-WFRFT- and SM-aided DM scheme is investigated. We assume the LUs know the SM mapping in advance and Eves cannot wiretap any number information. However, before performing the proposed scheme, we also need to exchange the MP-WFRFT parameters between the transmitter station and the LUs through a secure channel. In a practical application scenario, the Eve may know the MP-WFRFT modulation and intercept them with imperfect parameters. Therefore, it is necessary to analyze the effect of the leakage of MP-WFRFT parameters on the secrecy performance of our proposed scheme. Here, the actual MP-WFRFT parameters can be expressed as

(25) $$\begin{array}{c} \begin{array}{c} \left[\begin{array}{c} \hat{\alpha }\\ \hat{m}\\ \hat{c} \end{array}\right]=\left[\begin{array}{c} -\alpha_{s}\\ \;m_{s}\\ \;c_{s} \end{array}\right]+\left[\begin{array}{c} \Delta \alpha \\ \Delta m\\ \Delta c \end{array}\right] \end{array} \end{array}$$

where $\Delta \alpha ,\Delta m={\left[\Delta m_{1},\Delta m_{2},\Delta m_{3},\Delta m_{4}\right]}^{T}$, and $\Delta c={\left[\Delta c_{1},\Delta c_{2},\Delta c_{3},\Delta c_{4}\right]}^{T}$ are the mismatched errors. The detailed simulations and analysis are given in Section 5 for investigating the impact of these nine MP-WFRFT mismatched parameters on secrecy performance.

### 4.3. Discussion

In this section, we present a comparison with some previous works in Table 2, which fully compares different schemes from different aspects of power efficient (PE), neighbor security (the location of Eve is close to or the same as that of LU, NS), control of received power (CR), range-angle security (RA), and spectral efficiency (SE).

From Table 2, we generalize the advantages of our proposed scheme as follows.

(1) Compared with PA-based DM schemes, the proposed scheme can achieve the range-angle security due to the FDA characteristics.

(2) The high overall spectral efficiency. The idea of SM is to map a block of information bits to symbols that are chosen from the constellation diagram and numbers information of LUs that are chosen from the sets of LUs. The numbers information of multiple LUs can be directly used as additional sources to transmit information simultaneously.

(3) Compared with AN-aided DM schemes, our proposed scheme is power efficient due to MP-WFRFT technique which realizes the embedding process of “AN” from the modulation level of digital baseband signal.

(4) Our proposed scheme can guarantee security of confidential message in some challenging application scenarios. Based on conventional DM schemes, as long as an Eve is close enough to the LU, the confidential messages can be intercepted by the Eve due to constraint on beamwidth. To illustrate the advantage of the proposed schemes, we consider the worst case where Eves know the MP-WFRFT operation with perfect parameters, and these Eves with same number of LUs can cooperate with each other. Therefore, the received symbol vectors of Eves are first operated by the inverse MP-WFRFT with the shared parameters $\left(\alpha_{e},m_{e},c_{e}\right)$, which yields

(26) $$\begin{array}{cc} y_{E}\left(t\right) & =F_{\alpha_{e}}^{\left(m_{e},c_{e}\right)}\left(H_{E}^{H}\left(t\right)\mathrm{Dv}\left(t\right)\right)+F_{\alpha_{e}}^{\left(m_{e},c_{e}\right)}\left(n_{E}\left(t\right)\right)\\ & =H_{E}^{H}\left(t\right)DF_{\alpha_{e}}^{\left(m_{e},c_{e}\right)}F_{\alpha_{s}}^{\left(m_{s},c_{s}\right)}\left(u\left(t\right)\right)+F_{\alpha_{e}}^{\left(m_{e},c_{e}\right)}\left(n_{E}\left(t\right)\right) \end{array}$$

Here, we assume Eves know the MP-WFRFT operation with perfect parameters, which means

(27) $$\begin{array}{c} \begin{array}{c} \left(\alpha_{e},m_{e},c_{e}\right)=\left(-\alpha_{s},m_{s},c_{s}\right), \end{array} \end{array}$$

Then the (35) can be further written as

(28) $$\begin{array}{c} y_{E}\left(t\right)=H_{E}^{H}\left(t\right)DF_{-\alpha_{s}}^{\left(m_{s},c_{s}\right)}F_{\alpha_{s}}^{\left(m_{s},c_{s}\right)}\left(u\left(t\right)\right)+F_{-\alpha_{s}}^{\left(m_{s},c_{s}\right)}\left(n_{E}\left(t\right)\right)=H_{E}^{H}\left(t\right)\mathrm{Du}\left(t\right)+n_{E}^{{}^{\prime }}\left(t\right) \end{array}$$

Specifically, the received signal of jth Eve intercepting kth LU’s information can be obtained by

(29) $$\begin{array}{c} y_{E_{j}}\left(t\right)=h_{E_{k}}^{H}\left(t\right)Du_{k}\left(t\right)+n_{E_{j}}^{{}^{\prime }}\left(t\right)=\underset{\mathrm{Scrambled}\;\mathrm{signal}}{\underset{︸}{h_{E_{k}}^{H}\left(t\right)d_{k}u_{k}\left(t\right)}}+\underset{\mathrm{Interference}\;\mathrm{from}\;\mathrm{others}}{\underset{︸}{h_{E_{k}}^{H}\left(t\right){\sum_{i=1,i\neq k}^{K}d_{i}u_{i}\left(t\right)}_{i}}}+\underset{\mathrm{AWAG}}{\underset{︸}{n_{E_{j}}^{{}^{\prime }}\left(t\right)}} \end{array}$$

From (38), in the worst case, we can see that WFRFT operation cannot achieve encryption security. When the Eve’s location is the same as the LUs—i.e., $h_{E_{j}}\left(t\right)=h_{L_{k}}\left(t\right)$, $h_{E_{k}}^{H}\left(t\right)d_{k}=1$, $h_{E_{k}}^{H}\left(t\right)d_{i}=0\left(i\neq k\right)$—the AN–DM scheme and WFRFT-aided DM scheme both fail. However, based on our proposed scheme, it is also difficult for Eves to wiretap confidential messages, as estimates of the index information of LUs also need to be correct. Moreover, even if one or some (but not all) of the Eves’ locations are same as one or some LUs’ locations, the confidential messages are still difficult to retrieve for Eves. Therefore, our proposed method achieves better anti-interception performance and thus improves transmission security.

## 5. Numerical Results and Analysis

In this section, we provide several numerical experiments to evaluate the performance of the proposed SM and MP-WFRFT aided RFDA-DM scheme and compare its performance with conventional DM schemes. If not otherwise stated, our main parameters in the numerical experiments are given as Table 3.

### 5.1. Proposed Scheme Focusing Performance Analysis

As discussed in Section 3, the focusing of FDA-DM depends on range-angle dimensions, whereas the focusing of PA-DM only depends on angle. Therefore, our proposed scheme is based on FDA, and in order to improve the focusing performance, we propose random frequency offset and design the beamforming matrix D(t). Here, we give the spatial power distribution of confidential messages and the SINR spatial distribution to evaluate the focusing performance.

**Figure 5:**
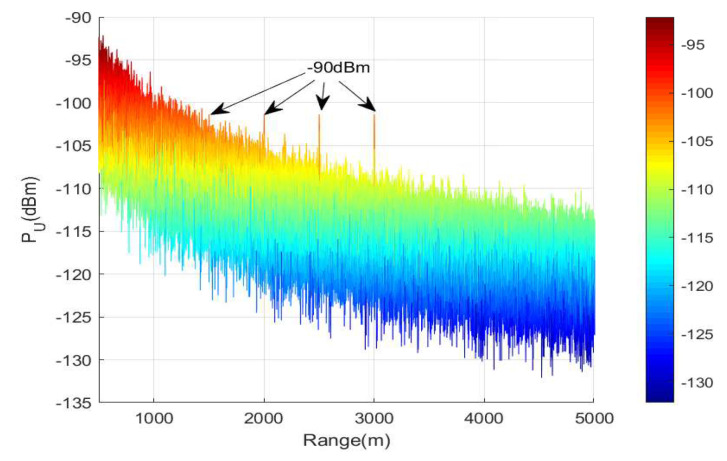
The spatial power distribution of confidential messages in range dimension.

The first experiment measures the energy distribution of confidential messages without the aid of SM and MP-WFRFT modulation. In this experiment, the minimum required receiving power of all LUs are set as $\sqrt{\varsigma_{1}}=\sqrt{\varsigma_{2}}=\sqrt{\varsigma_{3}}=\sqrt{\varsigma_{4}}=-90$ dBm. In Figure 5, it can be seen that the received confidential energy of LUs is almost equal to −90 dBm. Moreover, in the other region, the energy of confidential decreases as the distance increases. That means our proposed scheme can achieve confidential message energy focusing and accurate control the received power of each LU only based on RFDA.

**Figure 6:**
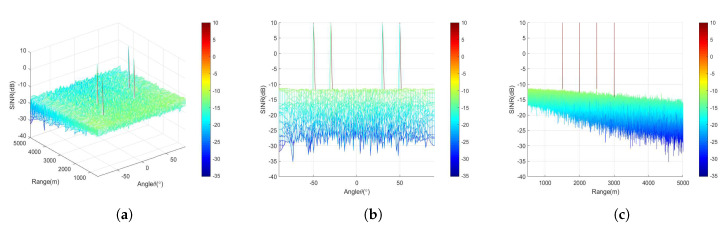
The signal to interference-plus-noise ratio (SINR) spatial distribution versus (**a**) angle-range, (**b**) angle dimension, and (**c**) range dimension.

In the second experiment, we simulate and analyze SINR distribution in free space. Here, we consider the case with independent LUs. The proposed approach combined with MP-WFRFT modulation to realize secure wireless communication. MP-WFRFT realizes the embedding process of “AN” from the modulation level of digital baseband signal. From Figure 6, it is obvious that there are four sharp peaks corresponding to each LU due to FDA with the design of beamforming matrix D(t) and the perfect inverse MP-WFRFT operation on received symbol vectors. Otherwise, the SINR of users in other regions is low, due to the weak received signal that is rotated by MP-WFRFT. In general, we achieve satisfactory focusing performance of wireless communication system.

### 5.2. Secrecy Performance Analysis

In the next experiments, we analyze the secrecy performance of our proposed scheme and compare it with the conventional AN-DM schemes. In a practical case, the Eves would be located as close to LU as possible (even in the same locations as LUs) to eavesdrop messages. We consider that there are four Eves whose locations are closed to LUs, even same as the LUs, and they cooperate with each other. The locations of Eves are set as $(r_{e_{1}}$, $\theta_{e_{1}})=(1600$ m, $30^{\circ })$ close to LU 1, $(r_{e_{2}}$, $\theta_{le_{2}})=(2000$ m, $-50^{\circ })$ same to LU 2, and $(r_{e_{3}}$, $\theta_{e_{3}})=(2500$ m, $55^{\circ })$ close to LU 3, $(r_{e_{4}}$, $\theta_{e_{4}})=(3050$ m, $-35^{\circ })$ close to LU 4. In order to show the effective reception of independent LU and verify the superiority of the SM with cooperative LUs, we first analyze BER performance of each LU (without cooperation) under different scenarios, and then simulate the SER performance of cooperative LUs.

**Figure 7:**
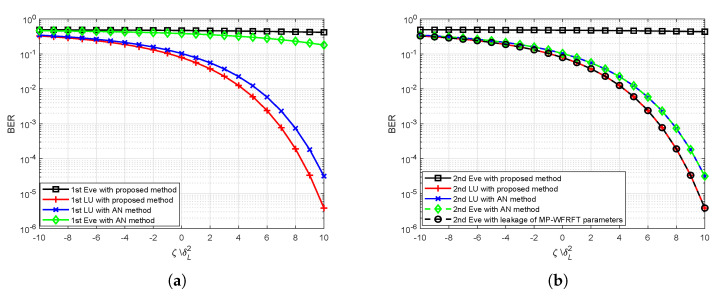
The bit error rate (BER) performance with independent LU versus the desired received power to noise ratio: (**a**) LU 1 and (**b**) LU 2.

Figure 7 illustrates the BER performance of LU and Eve versus the desired received power to noise ratio under different scenarios, where the baseband modulation modes of each LU is BPSK. From Figure 7a, we can observe that (1) our optimal method ensures the effective reception of LU 1; (2) the desired received power to noise ratio requested for the proposed scheme is less than the AN-DM schemes (approximately 1 dB when BER $\approx 10^{-2}$); (3) the BERs at Eve 1 are almost equal to $10^{-5}$ in Figure 7a, which means that an Eve close to a LU is difficult to receive meaningful message in proposed scheme and AN-DM schemes, respectively. LU 3 and LU 4 have a similar BER performance to LU 1. From Figure 7b, we can see that (1) LU 2 also has satisfactory BER performance; (2) even when the Eve’s location is the same as the location of LU 2, the Eve still cannot intercept the message transmitted to LU 2 with the proposed scheme, whereas the Eve successfully wiretaps the confidential message with AN-DM schemes; (3) in a worst case where MP-WFRFT parameters are perfectly leaked to Eves, our proposed scheme also cannot ensure secure transmission between transmitter station and LU 2.

**Figure 8:**
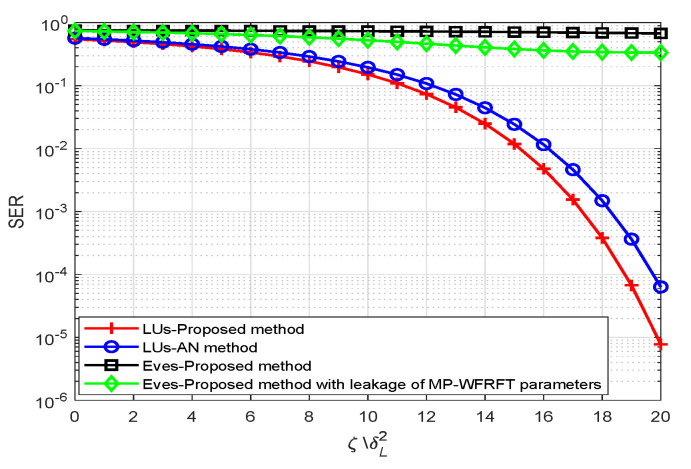
The SER performance of cooperative LUs versus the desired received power to noise ratio.

Next, Figure 8 shows the SER performance of cooperative LUs versus the desired received power to noise ratio, where the number of active LU is L=2. From Figure 8, we can observe that (1) the SER of Eves is low with all of desired received power to noise ratio, which shows that Eves cannot retrieve the confidential messages without/with the leakage of WFRFT parameters based on the proposed scheme. When MP-WFRFT parameters are leaked to Eves, the SER of Eves is only little better than the case without leakage; (2) the desired received power to noise ratio is less than the AN-DM schemes.

In general, the proposed scheme can ensure the valid reception for each LU. Moreover, with the SM and MP-WFRFT technology based on cooperative LUs, the eavesdropping capability of Eves is degraded, and thus we can ensure wireless communication security even in the worst case, in which one or some (but not all) independent messages and MP-WFRFT parameters are leaked to Eves. Furthermore, the WFRFT technique overcomes the low-power-efficiency drawback of the conventional AN-DM schemes, and the SM technology improves the capacity of communication system due to the use of indices information.

### 5.3. Anti-Interception Performance Analysis

**Figure 9:**
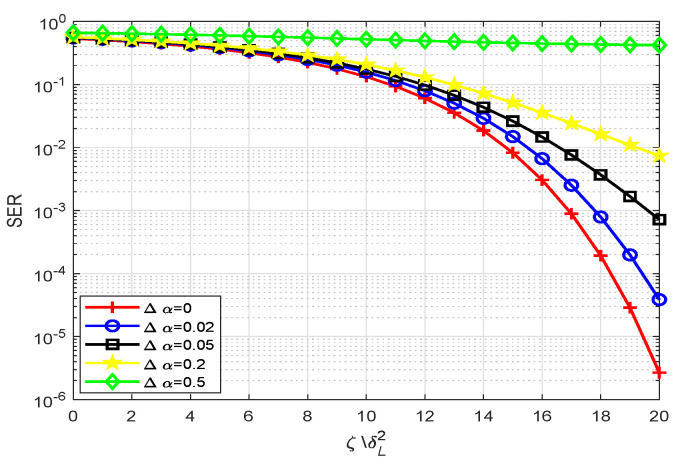
The anti-performance for proposed scheme on parameter α, where (m,c)=([0,0,0,0],[0,0,0,0]).

**Figure 10:**
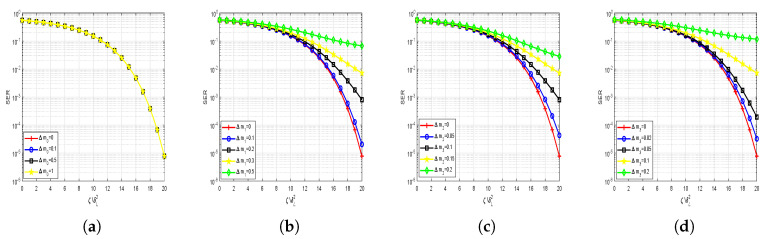
The anti-performance for proposed scheme on parameter m (**a**) $\left(\alpha ,m,c\right)=(0.5,\left[m_{0},0,0,0\right],\left[0,0,0,0\right])$, (**b**) $\left(\alpha ,m,c\right)=(0.5,\left[0,m_{1},0,0\right],\left[0,0,0,0\right])$, (**c**) $\left(\alpha ,m,c\right)=(0.5,\left[0,0,m_{2},0\right],\left[0,0,0,0\right])$, (**d**) $\left(\alpha ,m,c\right)=(0.5,\left[0,0,0,m_{3}\right],\left[0,0,0,0\right])$.

The anti-interception performance of our proposed scheme is depicted in Figure 9 and Figure 10, respectively. It is easy to see that the SER performance of the proposed scheme degrades a great deal along with some mismatched parameters. When the mismatch parameter Δα is small, the secrecy performance is almost unaffected, but the secrecy performance of the system declines rapidly with the increase of the Δα. When the mismatch parameter Δα widens to 0.5, the SER drops to about 0.5, which means that users cannot obtain any meaningful information. From Figure 10, we can conclude that $m_{k}$(k=1,2,3,4) have a similar but richer variation trend. Parameter $n_{k}$(k=1,2,3,4) have similar performance to $m_{2}$. Beside correctly estimating the LUs indices information, the eavesdroppers also need to meet the premise that nine parameters are completely consistent. Therefore, our proposed scheme has very good anti-interception performance even under the condition that the Eves know the signal transformation mode (SM modulation and WFRFT modulation).

## 6. Conclusions

In this paper, a security-enhanced and efficient multi-beam wireless communication scheme with cooperative LUs was proposed. In the proposed scheme, multiple important tools were utilized, including MP-WFRFT technology, SM modulation, and RFDA with the design of the beamforming matrix. With the help of MP-WFRFT, the scheme was found to be more power-efficient than conventional AN-DM schemes. Due to the SM technology, the proposed scheme was shown to have the ability to transmit information bits by the use of LUs number information apart from the modulation symbols. Finally, SINR distribution, BER performance and SER performance were simulated and analyzed, which verify the advantages of the proposed scheme.

## Figures and Tables

**Table 1:** SM map.

Bits Block	LUs Numbers	u(t)	Bits Block	LUs Numbers	u(t)
00000	1,2,3	[−1,−1,−1,0]	10000	1,3,4	[−1,0,−1,−1]
00001	1,2,3	[−1,−1,+1,0]	10001	1,3,4	[−1,0,+1,−1]
00010	1,2,3	[−1,+1,−1,0]	10010	1,3,4	[−1,0,−1,+1]
00011	1,2,3	[+1,−1,−1,0]	10011	1,3,4	[+1,0,−1,−1]
00100	1,2,3	[−1,+1,+1,0]	10100	1,3,4	[−1,0,+1,+1]
00101	1,2,3	[+1,−1,+1,0]	10101	1,3,4	[+1,0,+1,−1]
00110	1,2,3	[+1,+1,−1,0]	10110	1,3,4	[+1,0,−1,+1]
00111	1,2,3	[+1,+1,+1,0]	10111	1,3,4	[+1,0,+1,+1]
01000	1,2,4	[−1,−1,0,−1]	11000	2,3,4	[0,−1,−1,−1]
01001	1,2,4	[−1,−1,0,+1]	11001	2,3,4	[0,−1,−1,+1]
01010	1,2,4	[−1,+1,0,−1]	11010	2,3,4	[0,−1,+1,−1]
01011	1,2,4	[+1,−1,0,−1]	11011	2,3,4	[0,+1,−1,−1]
01100	1,2,4	[−1,+1,0,+1]	11100	2,3,4	[0,−1,+1,+1]
01101	1,2,4	[+1,−1,0,+1]	11101	2,3,4	[0,+1,−1,+1]
01110	1,2,4	[+1,+1,0,−1]	11110	2,3,4	[0,+1,+1,−1]
01111	1,2,4	[+1,+1,0,+1]	11111	2,3,4	[0,+1,+1,+1]

**Table 2:** Comparisons for different schemes.

ITEM	PA-DM	AN-DM	WFRFT-DM	PROPOSED-DM
PE	NO	NO	YES	YES
NS	NO	NO	YES	YES
CR	NO	NO	NO	YES
RA	NO	YES	YES	YES
SE	LOW	LOW	LOW	HIGH

**Table 3:** Simulation parameters.

Parameter	Value
Carrier frequency $f_{c}$	1 GHz
Number of FDA elements *N*	32
Inter-element spacing *d*	$c/2f_{c}$
Number of LUs *K*	4
Number of Eves *J*	4
Receive noise power,$10\log(\sigma^{2})$	−100 dBm
Location of LU1,$\left(r_{l_{1}},\theta_{l_{1}}\right)$	$\left(1500\;m,30^{\circ }\right)$
Location of LU2, $\left(r_{l_{2}},\theta_{l_{2}}\right)$	$\left(2000\;m,-50^{\circ }\right)$
Location of LU3, $\left(r_{l_{3}},\theta_{l_{3}}\right)$	$\left(2500\;m,50^{\circ }\right)$
Location of LU4, $\left(r_{l_{4}},\theta_{l_{4}}\right)$	$\left(3000\;m,-30^{\circ }\right)$
MP-WFRFT parameters (α,m,c)	(0.5,[1,2,3,4],[5,6,7,8])
